# Neuropsychiatric Manifestations and Cognitive Decline in Patients With Long-Standing Lyme Disease: A Scoping Review

**DOI:** 10.7759/cureus.58308

**Published:** 2024-04-15

**Authors:** Marissa Brackett, Jacklyn Potts, Allison Meihofer, Yumna Indorewala, Alina Ali, Sarah Lutes, Emma Putnam, Sophie Schuelke, Aisha Abdool, Emma Woldenberg, Robin J Jacobs

**Affiliations:** 1 Medicine, Dr. Kiran C. Patel College of Osteopathic Medicine, Nova Southeastern University, Fort Lauderdale, USA

**Keywords:** neuropsychiatric, cognitive decline, black-legged ticks, vector-borne disease, post-lyme disease syndrome, erythema chronicum migrans, lyme borreliosis, borrelia burgdorferi infection

## Abstract

Lyme disease (LD), or Lyme borreliosis, is a vector-borne disease that is caused by the transmission of the bacterium *Borrelia burgdorferi *through a tick bite. The symptoms of LD can persist in individuals chronically, even after the treatment and resolution of the initial infection. These symptoms include various neuropsychiatric manifestations and cognitive decline. The purpose of this review was to report the neuropsychiatric manifestations, cognitive decline, and effects of a delayed diagnosis on symptom severity in patients with long-standing LD (LSLD). A scoping review was conducted utilizing the electronic databases Embase, Ovid Medical Literature Analysis and Retrieval System Online (MEDLINE), and Web of Science. A total of 744 articles were retrieved and considered for inclusion. After a rigorous screening process, 10 articles that met the inclusion criteria for this review were included (i.e., reported neuropsychiatric manifestations and cognitive decline in patients with LSLD and the effects of a delayed diagnosis). Neuropsychiatric manifestations in the patients consisted of suicidal ideation, homicidal tendencies, extreme anger, depressive symptoms, aggression, and anxiety. Cognitive symptoms included dysfunctions in working memory, verbal learning/memory, non-verbal learning/memory, alertness, visuoconstructive, and frontal executive functioning. A delayed LD diagnosis increased symptom severity in most patients. The findings of this review indicate that neuropsychiatric and cognitive symptoms tend to present for a chronic period, even after disease recovery. Although researchers have established a link between a delayed LD diagnosis and increased symptom severity, LSLD is often an overlooked diagnosis in patients with neuropsychiatric symptoms and cognitive decline. More research is needed to compare the time to diagnosis and symptom severity in patients with LSLD.

## Introduction and background

Lyme disease (LD), or Lyme borreliosis, is an infectious disease transmitted to humans through a tick vector, with the responsible pathogen being *Borrelia burgdorferi*, a spirochete bacterium. In the United States, LD exhibits a bimodal age distribution, with spikes in prevalence observed among two distinct groups: children aged five to 15 years and adults aged 45-55 years [1]. Data suggest that there are 100 cases of LD per 100,000 people and 30,000 new diagnoses annually [2]. There are three clinically defined stages of LD. Stage 1 is considered the early localized stage, most commonly presenting with erythema migrans rash. Stage 2 is considered the early disseminated stage in which musculoskeletal, cardiovascular, and neurological symptoms begin. Stage 3, the late disseminated stage, presents with increased expression of these neurological manifestations. The late stage occurs when LD is left untreated and the bacteria proliferates in the central nervous system (CNS), causing significant neuropsychiatric symptoms and cognitive decline. This can include brain fog, schizophrenia, major depression, panic attacks, suicidal ideation, and obsessive-compulsive disorder [3]. There have also been instances where patients report symptoms long after treatment and disease resolution. As LD becomes increasingly prevalent, a better understanding of these comorbidities in patients with long-standing LD (LSLD) needs to be established. There is evidence of physicians failing to recognize the initial presentations of LD along with the later stage of the disease. A scoping review will help organize the current body of literature to explore the neuropsychiatric manifestations and cognitive decline found in patients with LSLD. This review will not explore any non-human subject research or ecology associated with LD.

Chronic LD (CLD) is defined as the presence of the symptomatology of LD continuously or intermittently for at least six months [4]. The term CLD has also been used as a nebulous term to describe either Lyme neuroborreliosis or late-stage disseminated LD. The ambiguous usage of this term has created confusion for the public and the medical field. This is an umbrella term that can be further broken down into two subcategories: 1) untreated CLD and 2) previously treated CLD [4]. Untreated CLD is delineated from previously treated CLD as a delay in diagnosis [4]. Furthermore, previously treated CLD has been referred to as post-treatment LD (PTLD), which refers to patients who have been treated for LD and still report neuropsychiatric and general inflammatory symptoms such as joint pain, muscle pain, and fatigue [4]. For this review, the anacronym LSLD is used to include all previously mentioned definitions.

Initially, LD is diagnosed based on the clinical presentation of the disease and/or a history of exposure to infected ticks. However, while erythema migrans rash is the most common presenting symptom, a retrospective study showed that it was absent in 13% of patients, and 54% of patients with the rash were still misdiagnosed [5]. Furthermore, this infection is generally seen by the public as a mild flu-like illness, requiring only a brief course of antibiotics [6]. In a qualitative study on the delay in the diagnosis and subsequent treatment of LD, patients cited nonspecific symptoms and the lack of a bulls-eye rash as reasons for not seeking treatment promptly [7]. On the physician side, a survey of 285 primary care physicians in Connecticut reported that 49.8% did not believe that LSLD existed and 48.1% were undecided on whether the condition existed [8]. If LD remains undiagnosed or untreated, it may progress to the more severe disseminated condition with neuropsychiatric and rheumatological manifestations that can become harder to accurately diagnose as a later stage of LD [8].

The psychiatric symptoms of LD are often misdiagnosed because researchers have only recently discovered the link between the two. In a retrospective cohort study, the accurate diagnosis rate of LD was found to be only 26.5% [9]. Neurological involvement was found in up to 40% of LD patients; meanwhile, depressive episodes occurred in up to 66% of patients [3]. The Infectious Diseases Society of America has discouraged clinicians from exploring the possibility of LD causing psychiatric illness, despite evidence to the contrary [10]. LD is commonly known as “the great imitator” because it is often an overlooked diagnosis and replaced with more common diseases. With the current literature available, the connection between neuropsychiatric symptoms and LD is becoming more apparent. Despite this, LD continues to be misdiagnosed with more familiar conditions.

No current or underway systematic or scoping reviews were found on a preliminary search of Medical Literature Analysis and Retrieval System Online (MEDLINE), the Cochrane Database of Systematic Reviews, and Joanna Briggs Institute (JBI) Evidence for neuropsychiatric manifestations and cognitive decline in patients with LSLD. While research has highlighted the connection between neuropsychiatric manifestations and cognitive decline in LSLD patients, there continues to be a gap in the knowledge of optimal management strategies and addressing these comorbidities. This review does not address all the gaps in the literature on treatments for LSLD patients with neuropsychiatric manifestations and cognitive decline. It does, however, highlight the associations between the lack of immediate treatment, prevalence, and the severity of these manifestations.

Objective

The purpose of this scoping review was to identify and characterize peer-reviewed studies published between January 1, 2013, and December 30, 2023, on the neuropsychiatric manifestations and cognitive decline found in patients with LD; address implications for delayed diagnoses in LD patients; assess potential groups that are high risk for developing neuropsychiatric symptoms in LD; and provide insight for future areas of research. Untreated LD in patients has important implications for public health, as well as healthcare, policy, and research. Although the literature has linked psychiatric comorbidities and cognitive decline with LSLD, there may be a gap in determining clinical treatment protocols.

## Review

Methods

This scoping review was conducted following the JBI methodology for scoping reviews.

Eligibility Criteria

Two tiers of this review were used to assess if the articles yielded from a systemized search using electronic scientific databases fit the inclusion criteria, which were based on the target patient population, the study design, and the study concept. Studies were included if they were original works published between January 1, 2013, and December 30, 2023, focusing on neuropsychiatric manifestations and cognitive decline found in patients with LSLD. Studies were also included if they focused on the delayed diagnosis of LD and the implications of delayed treatment. Studies were excluded if they were not in English; were dissertations, reviews, books, recommendations, opinions, policies, or guidelines; did not focus on the neuropsychiatric manifestations or cognitive decline found in LSLD patients; were published before 2013; and were not available as full text or were not published.

Search Strategy

The population, concept, and context (PCC) strategy was utilized for structuring the following research question: “In patients with long-standing Lyme disease (LSLD), what is the incidence of neuropsychiatric manifestations and cognitive dysfunction as a result of delayed treatment?” The population included patients with LSLD who had neuropsychiatric symptoms and/or cognitive dysfunction. The concept included the onset of various neuropsychiatric manifestations and cognitive dysfunction found in LSLD patients. The context included the prevalence of neuropsychiatric symptoms and cognitive dysfunction in patients who received a delayed LD diagnosis or treatment. The research team agreed on all terms utilized for the search, and one author conducted the search independently based on those terms before collecting the studies for the authors’ reviews. The following terms were used to search: “Lyme Disease” OR “Borrelia” AND “psychiatric symptoms” OR “depression” OR “anxiety” AND “delayed diagnosis” OR “delayed treatment.”

Information Sources

**Figure 1:**
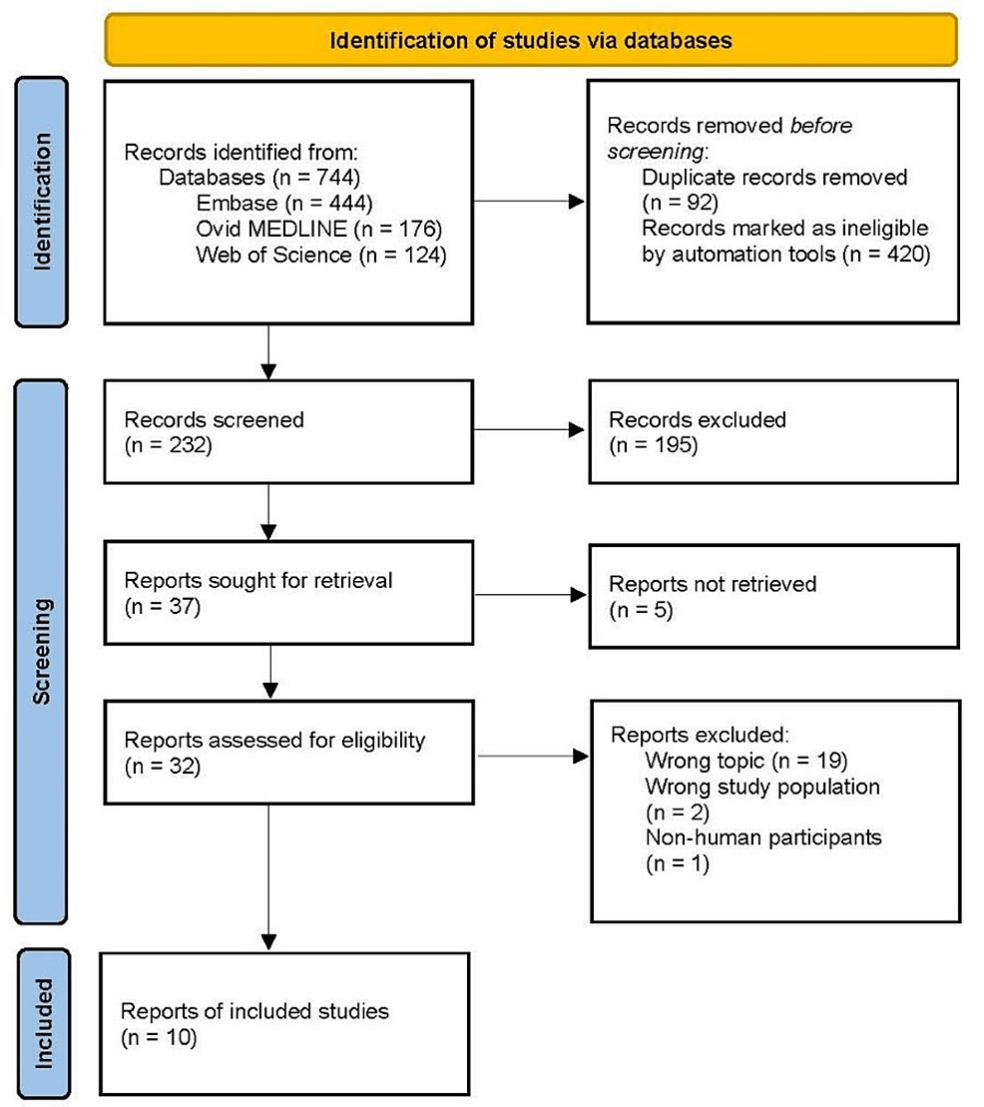
PRISMA Flow Diagram PRISMA, Preferred Reporting Items for Systematic Reviews and Meta-Analyses; MEDLINE, Medical Literature Analysis and Retrieval System Online

A search of the literature was conducted utilizing the Ovid MEDLINE, Embase, and Web of Science databases in October 2023. The initial search generated 744 articles. Ninety-two duplicate records were removed, and 420 records were marked as ineligible. As a result, 232 articles were left to be screened. In the first tier of the review, two authors analyzed the titles and abstracts of the articles for relevance to the review. The authors excluded 195 articles that did not meet the inclusion criteria. Thirty-seven articles were sought for retrieval, and five articles were excluded because the full text was not available. Three authors assessed 32 reports for eligibility in the second tier of the review. The authors excluded 19 articles because they did not evaluate the link between neuropsychiatric manifestations, cognitive dysfunction, and delayed diagnosis or treatment in LSLD patients. Two articles were excluded because they had the wrong study population, and one article was excluded because it involved non-human participants. In total, 10 articles were utilized in this scoping review. This screening and selection process is illustrated by a Preferred Reporting Items for Systematic Reviews and Meta-Analyses (PRISMA) flowchart (Figure 1).

Charting Process

The data charting process was conducted using Microsoft Excel (Microsoft Corp., Redmond, WA). Five reviewers extracted and charted data from each publication that was deemed relevant to our review (i.e., publication country of origin, study objectives/aims, study design, collection methods used, population(s) studied, sample size, key findings, and limitations).

Selection of Sources of Evidence

All the authors discussed the results and inclusion criteria for consistency before the initial screening of the 232 articles generated in the primary search. Two authors then worked independently to evaluate the abstracts and titles of the publications to determine their relevance to our study. Thirty-two full-text articles appeared relevant to the review and were further screened by three additional authors, resulting in 10 articles for the final review.

Results

The studies included were conducted in four countries: the United States (n = 5, three of which were conducted by the same author), Germany (n = 2), Poland (n = 2), and Belgium (n = 1). All studies included patients with a prior history of LD. A majority of the studies used questionnaires (n = 7) as part of the analysis. Three publications were retrospective studies, three were case-controlled studies, two were cohort studies, and two were cross-sectional studies. All studies were published between 2015 and 2022.

Neuropsychiatric Manifestations

Four out of 10 studies analyzed neuropsychiatric manifestations such as suicidal ideation, aggression, and mood disorders among those infected with LD [11-14]. One study found that suicidal ideation, homicidal tendencies, and extreme anger were reported in a significant number of LD patients compared to the general population [11]. Not only were suicidal tendencies more prevalent in those infected with LD, but also LD patients were found to be at a higher risk for developing extreme anger, social anxiety disorder, mood swings, hallucinations, and depression [11].

Patients with a prior history of LD frequently reported an increase in depressive symptoms post infection. One study found that 68% of patients with a current or prior diagnosis of LD had Beck Depression Inventory (BDI) scores that met the criteria for clinical depression [12]. Interestingly, another study from Poland found that patients who were diagnosed with early *B. burgdorferi* infection through serological testing presented higher levels of depression and anxiety compared to patients with late infection [13].

Aggression was also found to be common among those infected with LD. A pattern of impulsiveness followed by aggression was noted among these patients. This impulsiveness consisted of disorganized and intrusive thoughts, such as seeing images of death and pain [14]. Notably, temporal lobe dysfunction (which is associated with predatory aggression impairing the recognition of social emotions) was prevalent among patients, which resulted in partial seizures [14].

Cognitive Decline

Out of 10 articles analyzed, five found that generalized cognitive decline was evident among LD patients who had neuroborreliosis [12,15-18]. In comparison to healthy control populations, LSLD patients displayed deficits in working memory, verbal learning/memory, non-verbal learning/memory, alertness, visuoconstructive, and frontal executive functions. However, one study found that only the frontal executive portion was considered statistically significant [15].

Some of the most prominent clinical findings relating to cognitive dysfunction in LD patients post infection included sustained attention impairments, fatigue, brain fog, and impairments in memory [16]. One study found that LD patients performed worse on evaluations for alertness and memory compared to healthy control groups [12]. Only 46% of these studied patients received their diagnosis within the first five years after the development of their symptoms [12].

Historically, similar cognitive deficits have been recognized in other diseases such as major depressive disorder (MDD). However, Keilp et al. found that LSLD patients performed worse on tasks assessing verbal abilities, language fluency, working memory, and paragraph learning compared to MDD patients and healthy controls [17]. Interestingly, these cognitive deficits were found to disproportionately affect females. When compared to a group of males with neuroborreliosis, females with the same diagnosis demonstrated a significantly greater decrease in cognition [18].

Delayed Diagnosis and Symptom Severity

After LD treatment, many patients reported new or worsening neuropsychiatric manifestations, cognitive decline, and/or physical disability. Three studies indicated that the delayed diagnosis and treatment of LD were significant risk factors for developing LSLD and increased symptom severity [17,19,20].

Patients who were not treated proactively often reported continued or worsening symptom months to years after their initial diagnosis. One study found that LD patients who received delayed treatment reported higher levels of fatigue and muscle pain after a six-month follow-up compared to patients who were treated promptly [19].

One study found that 56% of LD patients who reported symptoms six months after treatment received a delayed or missed diagnosis [20]. These patients reported higher levels of pain, sleep quality, and depression compared to patients who received immediate treatment [20]. Another study found that LD patients with a delayed diagnosis had higher BDI scores compared to the general population [17]. Scores for working memory and verbal comprehension were also found to be significantly lower than healthy control groups [17]. Table 1 summarizes the 10 articles in this review.

**Table 1:** Summary Table of the Included Articles LSLD, long-standing Lyme disease; ELISA, enzyme-linked immunosorbent assay; CSF, cerebrospinal fluid; MMSE, Mini-Mental State Examination; CDT, clock drawing test

Reference	Country	Study Objectives/Aims	Study Design	Sampling Data/Collection Methods	Sample	Key Findings	Limitations
Bransfield (2017) [11]	United States	To investigate the association between suicide, Lyme, and associated diseases	Retrospective study: clinical chart review of patients with Lyme-associated diseases (LAD). Measured prevalence of suicidal and homicidal tendencies among these patients	Randomly selected inactive patient charts with LAD from the author’s practice were analyzed. Four groups based upon symptoms were made: suicidal and homicidal, suicidal not homicidal, experiencing explosive anger not suicidal or homicidal, and not suicidal, homicidal, or experiencing explosive anger	Two hundred fifty-three patients with LAD (58% female, 42% male, ages 8-64 years, and average age of 39 years). Clinical or serological Lyme disease (LD) diagnosis	Participants were infected an average of eight years before diagnosis and antibiotic treatment, demonstrating a delayed diagnosis. Forty-three percent had suicidal tendencies. Twenty-six percent of these suicidal patients were also homicidal. However, there were no patients who were homicidal without also being suicidal	The chart review included patients who did not meet the CDC criteria-based definition for LD (two-tier testing). Researchers calculated the rate of suicide based on a CDC calculator and patient symptoms. Patients who were co-infected with other viruses were included
Hündersen et al. (2021) [12]	Germany	To examine the relationship between neuropsychiatric and psychological symptoms in patients with Lyme borreliosis (LB)	Online questionnaire via survey software (Unipark, Tivia, New York, NY). Observational cross-sectional study February-April 2020. Compared experimental group (LD patients) to a control group	Researchers collected data from both the experimental group and control group via an online questionnaire that was shared via social media (Facebook) and flyers in medical facilities and through the test person system of the Medical School of Hamburg. The questionnaire assessed the individuals’ quality of life, sleep quality, attention, depressive symptoms, memory, and careers	The experimental group (history of LD): 189 females, 62 males, and one non-binary, average age of 51 years (range: 19-84 years); control group: 211 females and 56 males, average age of 27 years (range: 18-70 years)	Lyme disease patients often wait an average of eight years and see an average of eight physicians before a final diagnosis is made. Less than half the subjects (46%) received their diagnosis within the first five years after the development of symptoms. Results showed that LD can cause limitations in the quality of life, sleep, attention, memory, and depressive symptoms. Statistically significant differences were observed in the prevalence and severity of these symptoms when compared to the control group	Data were self-reported. One diagnostic test included in the questionnaire for diagnosing LD was lymphocyte transformation test (LTT), which is not recommended due to its low specificity
Makara-Studzinska et al. (2017) [13]	Poland	To evaluate the symptoms of depression and anxiety associated with fibromyalgia and erythema migrans (EM) in *Borrelia burgdorferi* infection	Case-by-case study. Measured levels of anxiety and depressive symptoms among patients diagnosed with *B. burgdorferi* infection who also reported symptoms associated with fibromyalgia. Questionnaires and serological tests were used to gather data	A group of 87 patients with clinically and serologically diagnosed *B. burgdorferi* (mean age of 53.37) was formed. Subjects were examined using the State-Trait Anxiety Inventory, Beck Depression Inventory (BDI), various immunological tests, and tests for fibromyalgia	Eighty-seven participants (43 females and 44 males) diagnosed and treated for *B. burgdorferi* infection who also presented with symptoms of fibromyalgia	Patients with early *B. burgdorferi* infection also diagnosed with fibromyalgia showed the highest levels of anxiety and depression. The results showed that serological markers of infection could act as indicators of depression level in patients with *B. burgdorferi* infection. Patients with IgM- and IgG+ serological markers, showing more advanced infection, had the lowest levels of anxiety and depressive symptoms	All patients were ethnically white Europeans. The sample size of this study is small, and other studies have shown that IgG antibody levels do not differ between healthy and infected people in regard to depression symptoms
Bransfield (2018) [14]	United States	To examine the association between aggressiveness, homicidality, homicide, and LD	Retrospective study design, measured aggressive behaviors before and after LD diagnosis compared to a control group via clinical chart reviews	Patient charts were reviewed and analyzed. Fifty patients with previous homicidal history and 50 patients with no homicidal history were included. Aggressive behavior rate was compared before LD infection to after within each group	One hundred total charts reviewed (50 homicidal and 50 non-homicidal LD patients); 52% male and 48% female	Of the LD patients, 9.6% were homicidal with an average diagnosis delay of nine years. Most aggression in LD patients was impulsive, usually provoked by intrusive symptoms, sensory stimulation, or frustration	The first chart review conducted included 1,000 charts from LD patients, and 50 were reported to show homicidal tendencies
Schmidt et al. (2015) [15]	Germany	To determine the frequency and extent of changes in cognitive function in patients with neuroborreliosis (NB)	Case-controlled study; measured brain atrophy and cognitive decline using clinical workups and questionnaires	Sixty patients were given neurological and neuropsychological workups six months or longer after treatment for proven NB. Standardized neurological examinations quantified with the Scripps Neurological Rating Scale (SNRS) were used to assess cognitive function. The results were compared to a group of 30 healthy controls who tested negative for *B. burgdorferi*	Sixty patients with neuroborreliosis between ages 15 and 70 at the time of treatment. Thirty patients who tested negative for *B. burgdorferi*	Patients in the NB group showed lower scores on the SNRS and lower performance in frontal executive functions compared to the healthy control group. Cognitive sum scores were also lower in the NB group compared to the control group. There were no significant differences found for health quality of life, sleep disturbance, psychiatric symptom load, or brain atrophy	No major limitations noted
Bransfield et al. (2020) [16]	United States	To describe the clinical presentation of Lyme borreliosis patients with chronic, late-stage psychiatric symptoms from a review of 100 charts and to develop a clinical assessment system from these findings	Observational retrospective cohort study. Questionnaire used to compare patient’s symptoms pre-infection and post infection	The first author developed the assessment form used in this study to evaluate patients with late-stage LD. The same assessment was performed on all patients by the first author. The date of infection was also established from each chart	One hundred patients enrolled; the average age was 38 years (range: 6-89 years)	Out of 100 patients enrolled, 70 had a delayed diagnosis and treatment, with the average delay being nine years. The prevalence of each clinical finding pre-infection and post infection was compared and calculated within the 95% confidence interval. The average patient had five symptoms pre-infection and 82 post infection	The patient’s neuropsychiatric status before infection was approximated based on recall
Keilp et al. (2019) [17]	United States	Patients with post-treatment Lyme disease syndrome (PTLDS) were compared to patients with major depressive disorder (MDD), as well as healthy comparison subjects, to see if there were significant differences in cognitive function between the two groups	Cohort study; measured cognition, including memory in patients with MDD and PTLDS via clinical workups and questionnaires	Performance on Wechsler Adult Intelligence Scale-III (WAIS-III) and Wechsler Memory Scale-III (WMS-III) on subgroups of PTLDS, MDD, and healthy controls	Ninety-seven healthy controls (average age of 38.3); 81 PTLDS (average age of 47.8); 92 MDD (average age of 38.9)	Most patients in the PTLDS group had a relatively late diagnosis after initial symptom onset, thus leading to a relatively long period of illness before initial treatment. Although mean scores on most WAIS-III and WMS-III tests in the PTLDS group did not fall outside of the “average” range, they reflect consistently lower performance on measures of working memory, learning, and information retrieval in a sample with above-average ability. BDI scores in LSLD patients were statistically higher than those found in the healthy population but lower than those diagnosed with MDD	No major limitations noted
Oczko-Grzesik et al. (2017) [18]	Poland	To assess the functional status of patients with borreliosis and NB in relation to the frequency of the occurrence of cognitive deficits, depression, and anxiety disorders	Cross-sectional study, 2005-2012. Compared patients with Lyme arthritis and Lyme NB through clinical workups, interviews, and laboratory testing. The outcome measures included cognitive functioning and affective functioning	Data was collected from patients hospitalized at the Department of Infectious Diseases, Specialistic Hospital No. 1, in Bytom, Poland, between 2005 and 2012. The data collection methods included a standardized interview, clinical examination, laboratory tests (ELISA, western blot, and CSF), and psychological testing (MMSE, CDT, and BDI)	One hundred twenty-one patients (61 females and 60 males), ages 18-65 (average 40 years), who were diagnosed with late-stage LD (mean duration of 2.5 years). There were 46 patients (31 females and 15 males) with Lyme arthritis and 75 patients (30 females and 45 male) with Lyme NB	In patients with LD, particularly in those with NB, an increased frequency of depressive and neurotic disorders was observed. Neurotic disorders, mainly adaptive, are most common in males with LD, while depressive disorders were more frequent in females. In patients with NB, particularly in females, an increased frequency of cognitive deficits was observed. After antibiotic therapy, the prevalence of depression, anxiety, and mild cognitive disorders decreased in patients with Lyme arthritis and NB	No major limitations noted
Geebelen et al. (2022) [19]	Belgium	To compare nonspecific symptoms between Lyme borreliosis (LB) patients and a non-LB control at six- and 12-month follow-up; to estimate the proportion of PTLDS in two groups of LB patients: patients with erythema migrans (EM) and disseminated or late LB	Prospective cohort study, from June 2016 to December 2019. Compared nonspecific symptoms among patients diagnosed with EM to disseminated/late LB and to a non-LB control group using questionnaires	LB patients were asked to fill in a questionnaire at diagnosis at one, three, and six months (12 and 24 months if possible) post treatment. These questionnaires were also completed by the control group at the time of inclusion, six months after inclusion, and 12 months after inclusion	Participants were 18 years or older; 120 patients with EM (46 males and 74 females); 15 patients with disseminated/late LB (13 males and two females); 128 healthy control patients (46 males and 82 females)	The proportion of PTLDS patients were found to be 5.9% (95% CI: 2.7-12.9) in EM patients and 20.9% (95% CI: 6.8-64.4) in disseminated/late LB patients. The chance of receiving a PTLDS diagnosis was found to be significantly higher in patients with late or disseminated LB	Predefined sample sizes could not be included; small sample size for the disseminated/late LB group; the disseminated/late LB group was heavily skewed toward males
Rebman et al. (2017) [20]	United States	To delineate PTLDS-specific patterns in physical examination findings, clinical laboratory results, symptom reporting, and quality of life. These patterns would then be compared to control participants with no prior history of LD	Case series style: outcome measures were the quality of life based on a 36-item symptom list. Patients were evaluated through clinical workups and questionnaires	The participants with PTLDS were physician- or self-referred to the Johns Hopkins Lyme Disease Research Center. A clinical, laboratory evaluation and a quality of life analysis were conducted on the patients. Symptoms were measured by standardized questionnaires including the Fatigue Severity Scale (FSS), the short-form McGill Pain Questionnaire (SF-MPQ), and the BDI	Sixty-one participants met the criteria for PTLDS (52.5% female, 93.4% white and non-Hispanic); 26 participants had no prior history of LD or PTLDS and served as healthy controls (53.9% female, 88.5% white and non-Hispanic)	Initial delayed or misdiagnosis was characterized in 59% of the participants with PTLDS. Patients in the PTLDS group were highly symptomatic, with poor health-related quality of life. PTLDS patients exhibited higher levels of fatigue, musculoskeletal pain, sleep disturbance, and depression compared to the healthy control group	No major limitations noted

Discussion

LSLD has been described by scientists and physicians as a new, continuing, or worsening symptoms for at least six months after treatment for LD infection. Many patients who received treatment for LD reported new or worsening neuropsychiatric manifestations and/or cognitive decline. If treatment for LD is delayed, neuropsychiatric symptoms and cognitive decline often worsen and frequently lead to the development of LSLD [17,19,20].

Neuropsychiatric Comorbidities and Cognitive Decline

Different neuropsychiatric manifestations have been associated with prior exposure to LD. Depressive symptoms and suicidal tendencies were among the most common manifestations reported, although there was evidence for increased anxiety, fear, anger, neuropathy, and mood swings [11,16,17,20]. Patients who had previous exposure to LD reported a higher incidence of depressive symptoms than those who had never been exposed to the infection [17]. BDI scores in LSLD patients were statistically higher than those found in the healthy population but lower than those diagnosed with MDD [17]. This suggests that some people who have LSLD do not meet the criteria to be diagnosed with MDD but experience a higher level of depressive symptoms than the general population. Interestingly, one study reported that patients in the later stages of LD who had IgG antibodies showed lower levels of depression and anxiety compared to those in the earlier stages of the disease [13]. However, it should be noted that other studies have shown that IgG levels do not correlate with depression severity in the general population.

In addition to depressive symptoms, there appears to be an increased risk for suicidal tendencies in patients with LSLD. This may be due to changes in immune- and metabolic-mediated pathways, which can lead to an increased prevalence of psychiatric symptoms including paranoia, mood swings, dissociative episodes, and substance use disorders [11]. The increase in mental health struggles along with the increased incidence of depression and depressive symptoms can increase the risk of suicidal ideation and suicide attempts. Additionally, there is a stigma surrounding LSLD because it is difficult to diagnose. Many patients are often diagnosed with other conditions that have similar symptoms, causing them to feel dismissed by their physicians. These patients may have negative experiences with healthcare providers, family, or friends, which could lead to increased feelings of sadness and anger.

Sleep difficulty was reported as one of the most prominent features of LSLD. Sleep difficulties can lead to extreme fatigue, which could be a contributing factor to other neuropsychological symptoms and cognitive decline. The Pittsburgh Sleep Quality Index (PSQI) scores in LSLD patients were statistically lower than those found in healthy individuals [20]. It has been hypothesized that a decrease in sleep can lead to other symptoms such as depression, visual disturbances, and musculoskeletal pain [20]. Additionally, memory and processing difficulties were commonly reported leading to spelling mistakes, reversals of numbers and letters, and decreased fluency in language or speech [16]. A mild to moderate decline in memory and processing speeds has also been associated with LSLD [17]. This decrease in sleep quality could be implicated in the various neuropsychiatric symptoms and cognitive dysfunctions found in LSLD patients.

Delayed Diagnosis and Severity of Symptoms

It can be difficult for some physicians to diagnose and treat LD patients early because it can present with a multitude of nonspecific symptoms [12]. There are no clearly defined diagnostic criteria for diagnosing LD due to the wide array of presentations, which makes early diagnosis and treatment difficult [16]. It may be prudent for physicians to diagnose and treat this disease in its earlier stages to prevent more severe neurological, neuropsychiatric, cardiological, and musculoskeletal symptoms from developing [12]. It is also important to note that if a patient lives or has traveled to an endemic area and is experiencing neuropsychiatric and cognitive changes, LSLD should be ruled out. When treated early, neuropsychiatric deficits and cognitive decline are much less common and can have a smaller impact on the patient’s quality of life [17].

Lack of Public Health Awareness

Public health reports provide limited information on the neuropsychiatric comorbidities of LD. Utilizing a mixed-method approach, Maxwell et al.’s study triangulates three data sources to compare official public health information, case reports, and medical literature to self-reported symptoms in patients with LD [21]. Among the 15 neuropsychiatric symptoms documented in the medical literature for LD, Maxwell et al. found that public health officials only fully recognized headaches and fatigue [21]. Additionally, LD is the least acknowledged tick-borne illness by public health officials for its neuropsychiatric symptoms.

Given the plethora of nonspecific patient symptoms and the diverse neuropsychiatric presentations not in line with public health guidance, this study underscores the necessity for a revised approach to LD and improved communication from official public health sources regarding the broad spectrum of associated symptoms. Hikers in endemic areas should be made aware of prevention methods such as mosquito nets and sprays, as well as the various presentations of LD infection. Outdoor enthusiasts should check their bodies for signs of an erythema migrans rash so as to obtain prompt treatment. Similarly, physicians are encouraged to thoroughly evaluate patients who present with symptoms of LD to prevent a delay in diagnosis.

Limitations

This is the first scoping review to our knowledge to explore the various neuropsychiatric manifestations and cognitive dysfunctions found in patients with LSLD, including the effects of a delayed diagnosis on symptom severity. One limitation of the articles in this review was the various definitions used for patients with LSLD. These included “chronic LD” and “post-treatment LD,” making it challenging to categorize and collate the information provided. Some studies had small sample sizes, and the study groups lacked diversity [13,19]. In one article, some patients included in one study had co-infections with other diseases [11]. In another study, patients lacked definitive knowledge regarding their pre-LD neuropsychiatric status [16]. Most patients who had a delayed LD diagnosis and treatment acquired LSLD and increased symptom severity, but no studies compared the time to diagnosis with symptom severity. Another study included patients who did not meet the CDC criteria for LD [11]. In addition, questionnaires were a common source of data for the majority of the articles, which can be subjective (e.g., patients under- or overreporting symptoms). Since patients with LD are often more active pre-infection (hikers and outdoors enthusiasts), these patients may report a lower quality of life post infection because they are not able to be as active as they were pre-infection. Moreover, the articles from Europe [12,13,15,18,19] reported very different findings compared to those from the United States [11,14,16,17,20]. This could be due to the different strains of *Borrelia* found in the United States and Europe. Due to these limitations, the findings should be interpreted with caution.

Implications for Future Research

Future research is needed to determine the exact pathophysiology behind the development of neuropsychiatric symptoms and cognitive decline in patients with LSLD and how these manifestations can persist for years. Further studies should be conducted to compare LD and LSLD presentations in patients in and outside the United States. More objective studies, such as physical examinations or brain imaging, are needed to determine the similarity between the pathophysiology of depression, fatigue, and LD. Moreover, there is a need for prospective and comparative studies. Additionally, more rigorous studies need to be conducted exploring the connection between time to diagnosis and symptom severity, as no reports with specific data defining the period of time it takes for LD infection to progress into LSLD were found.

## Conclusions

This scoping review summarized the available literature on LD, neuropsychiatric manifestations, and cognitive decline. Implications for delayed diagnoses in LD patients and possible future areas of research were discussed. LD can result in many neuropsychiatric manifestations and cognitive decline, and these symptoms can appear or worsen months to years after treatment. The delayed diagnosis of LD can result in an increased risk of developing LSLD and exacerbated symptom severity. Moreover, depression, suicidal tendencies, neuropsychiatric conditions, and cognitive decline have been linked to prior infection with LD. Diagnosing and treating LD in its early stages can help prevent the development of LSLD. While the articles in this review suggest neuropsychiatric manifestations and cognitive decline with a delayed LD diagnosis, more rigorous research needs to be conducted to determine the pathophysiology behind LSLD and why these symptoms become chronic in some patients.
